# Measles outbreak investigation in Zaka, Masvingo Province, Zimbabwe, 2010

**DOI:** 10.1186/1756-0500-5-687

**Published:** 2012-12-19

**Authors:** Kufakwanguzvarova W Pomerai, Robert F Mudyiradima, Notion T Gombe

**Affiliations:** 1 Department of Community Medicine, University of Zimbabwe, P. O. Box A178, Harare, Zimbabwe; 2 Provincial Medical Director Masvingo Province, Masvingo, Zimbabwe

**Keywords:** Measles, Outbreak, Risk factor, Zimbabwe

## Abstract

**Background:**

A measles outbreak was detected at Ndanga Hospital in Zaka district Masvingo Province on the 5th of May 2010 and there were five deaths. Source of infection was not known and an investigation was carried out to determine factors associated with contracting measles in Zaka district.

**Materials and methods:**

A 1:1 unmatched case control study was conducted. A case was a person residing in Zaka district who developed signs and symptoms of measles or tested IgM positive from 06 May 2010 to 30 August 2010. A control was a person residing in the same community who did not have history of signs and symptoms of measles during the same period. A structured interviewer administered questionnaire (translated into shona) was used to solicit information from cases and controls. Ethical consideration like written consent from all participants, respect and confidentiality were observed. Permission to carry out the study was obtained from the medical research Council of Zimbabwe and the provincial Medical Directors Masvingo. Epi info was used to calculate frequencies, odds ratios and perform logistic regression to control for confounding variables.

**Findings:**

A total of 110 cases and 110 controls were recruited. Most cases (63.03%) were from the apostolic sect while 44.7% of controls were from orthodox churches. Contact with a measles case [AOR= 41.14, 95% CI (7.47-226.5)],being unvaccinated against measles [AOR= 3.96, 95%CI (2.58-6.08)] and not receiving additional doses of measles vaccine [AOR 5.48, 95% CI (2.16-11.08)] were independent risk factor for contracting measles. Measles vaccination coverage for Zaka district was 75%. The median duration for seeking treatment after onset of illness was three days (Q1=2; Q3=7). There were no emergency preparedness plans in place.

**Conclusion:**

This outbreak occurred due to a large number of unvaccinated children and a boarding school that facilitated person to person transmission. We recommend mandatory vaccination for all children before enrolling into schools. As a result of the study one day training on outbreak management and surveillance was done with all District Nursing Officers and Environmental Health Officers in personnel in the province.

## Background

Measles is a very infectious viral disease that affects children below the age of 15 years. The signs and symptoms of measles include fever, lack of appetite, cough, coryza, red eyes, and maculopapular rash, with complications such as pneumonia, blindness, brain damage, diarrhoea and croup. The incubation period of the disease ranges from 10 days to a month [1]. Measles is spread through contact with nose and throat secretions of infected people and through airborne droplets released when an infected person sneezes or coughs. A person can infect others for several days before and after he or she develops symptoms. The disease spreads easily in areas where infants and children come into contact such as in health centres and schools [2].

Measles remains the leading cause of childhood morbidity and mortality in the world. Globally, more than 20 million cases are reported yearly and 345 000 deaths were recorded in 2005. Fifty to sixty percent of 1.6 million global deaths attributed to vaccine preventable diseases are attributed to measles [3]. In Africa 450 000 cases were reported and in Sub Saharan Africa 250 000 deaths were reported in 2009 [3]. In 2009, Zimbabwe reported 1200 cases of measles. Between May and August 2010 Masvingo province reported 126 cases from Zaka district. Measles is one of the vaccine preventable diseases that are targeted for elimination and with half of the world close to eliminating measles, many countries in sub Saharan Africa including Zimbabwe are still struggling to control the disease [4].

Due to an increase in vaccination coverages in developing countries there has been a significant change in the epidemiology of measles such as higher incidence in older children and young adults [5]. Under nourished people are more susceptible to measles complications, slow recovery, and higher fatalities. Being vaccinated against measles gives protection against measles up to 99% and the World Health Organisation recommends that all children who receive the first dose of vaccine should also have a second opportunity for vaccination [6]. In Zimbabwe the Ministry of health and Child Welfare recommends immunisation against measles to be at nine months since vaccinating earlier than 9 months affects the potency of the vaccine due to interaction with maternally acquired antibodies [2]. High coverages of vaccination of children below the age of 15 years has led to reduction of measles cases by up to 99% in developed or industrialised countries [7]. Developing countries are failing to achieve high vaccination coverages, hence frequent outbreaks of measles with high case fatalities as high as 3- 30% [7].

Zaka district experienced a measles outbreak with a total 126 cases as at 30 August 2010. Five cases were IgM positive. The index case was a 29 year old man with signs and symptoms of measles from Ndanga who reported at Ndanga Hospital on the 6th of May 2010. Further presentation of patients with similar symptoms and reports from villagers led to the declaration of a measles outbreak. An investigation of the outbreak was conducted to determine factors associated with contracting measles in Zaka district as well as to assess the district’s preparedness and response to the outbreak.

The null hypothesis for the study was; there is no association between demographic, socio economic and socio cultural factors and contracting measles in Zaka district.

The alternative hypothesis was: there is an association between demographic, socio economic and socio cultural factors and contracting measles in Zaka district.

## Methods

A 1:1 unmatched case control study was conducted in Zaka district. A case was any person who resided in Zaka district who developed any of the following symptoms; fever, lack of appetite, cough, coryza, red eyes, maculopapular rash or tested IgM positive between 06 May 2010 and 30 August 2010. A control was any person who resided in the same community or village with cases in Zaka district who did not have history of signs and symptoms of measles or tested IgM negative between 06 May 2010 and August 30, 2010.

### Inclusion criteria

#### Cases

Any resident of Zaka district who tested positive for IgM or had symptoms of measles from 05 May to 30 August 2010 and who agreed to participate in the study was included.

#### Controls

A control was any resident of Zaka district during the study who was a neighbour to a case and who did not develop signs and symptoms of measles and agreed to participate was included.

### Exclusion criteria

#### Cases

Those who refused to participate or were unconscious were excluded.

#### Controls

Those who refused to participate were excluded as well as family members from the same house hold.

The sample size was calculated using Stat calc function of Epi-info version 3.3.2. Using the confidence level of 95%, power of 80%, and assuming a 36.7% prevalence of a previous contact with someone with a measles like disease in under ones [8] and an OR 3.64, 101 cases and 101 controls were required.

Cases were selected randomly using the lottery method where each name on the line list was allocated a number on pieces of paper which were put in a box and randomly picked and the name corresponding to that number was recruited into the study. The picked number was replaced and the process started all over again until the sample size was reached.

Controls were neighbours of cases who did not suffer from measles during the period of the study. Only one control for one case per house hold was selected from the neighbours of cases.

A invalidated structured interviewer administered questionnaire (back translated in to shona the local language) was used to collect data on factors associated with contracting measles, community knowledge and practices on measles for both cases and controls.

Review of cases notes was done to assess case management (treatment given to patients) and road to health cards checked to verify immunisation date, batch numbers and vitamin A supplementation. A checklist was used to assess the district’s preparedness and response.

Permission to carry out the study was obtained from the Medical Research Council of Zimbabwe, Provincial Medical Director Masvingo(PMD), District Medical Officer(DMO) Zaka, and Health Studies Office(HSO).

An informed written consent was obtained from all study participants. Confidentiality was assured throughout by not writing participants names. Participants were treated with respect and willingly participated in the study with no payment or cohesion. Permission to take photographs was obtained from parents or guardians for minors below 16 years while participants above 16 years were asked for their own consent. Questionnaires were translated into local language.

## Results

A total of 110 cases and 110 controls were recruited into the study. Participants were comparable in almost all demographic characteristics except for age as shown in Table 1.

**Table 1:** Demographic characteristics of measles cases and controls, Zaka District, Masvingo Province, 2010

**Variable**		**Cases n= 110 (%)**	**Controls n= 110 (%)**	**P value**
Median Age		10 years(Q1=7,Q3=16	16 years(Q1=10,Q3=19)	0.04
Sex	Females	65(59.1%)	65(59.1%)	0.11
	Males	45(49.1%)	45(40.9%)	
Marital Status	Single	107(97.3%)	93(84.5%)	0.90
	Married	3(2.7%)	17(15.5%)	
Religion	Apostolic	70(63.6%)	33(30.00%)	0.10
	Orthodox	24(21%)	47(44.7%)	
	Pentecostal	15(13.6%)	28(25.5%)	
	Traditional	1(0.9%)	2(1.8%)	
Occupation	Formally employed	1(0.9%)	4(3.6%)	0.35
	School Pupil	86(78.2%)	82(74.5%)	
	Unemployed	23(20.9%)	26(23.6%)	
Level of Education	None	24(21.8%)	10(9.1%)	0.06
	Primary	39(35.5%)	33(30.0%)	
	Secondary	47(42.7%)	67(60.9%)	
Median Number of people per House		9(Q1=6),(Q3=14)	6(Q1=5) ,(Q3=10)	0.08
Median Number of Siblings Sharing Room		4(Q1=3),(Q3=7)	3(Q1=2),(Q3=4)	0.70

Of the 126 cases reported on the line list, all five specimens collected tested IgM positive and 5 community deaths were reported giving a case fatality rate of 4%.Three of the five deceased cases were males, all of them were unvaccinated, and all five cases belonged to the apostolic sect. The major complications reported by the deceased were diarrhoea, cough and croup.

**Figure 1:**
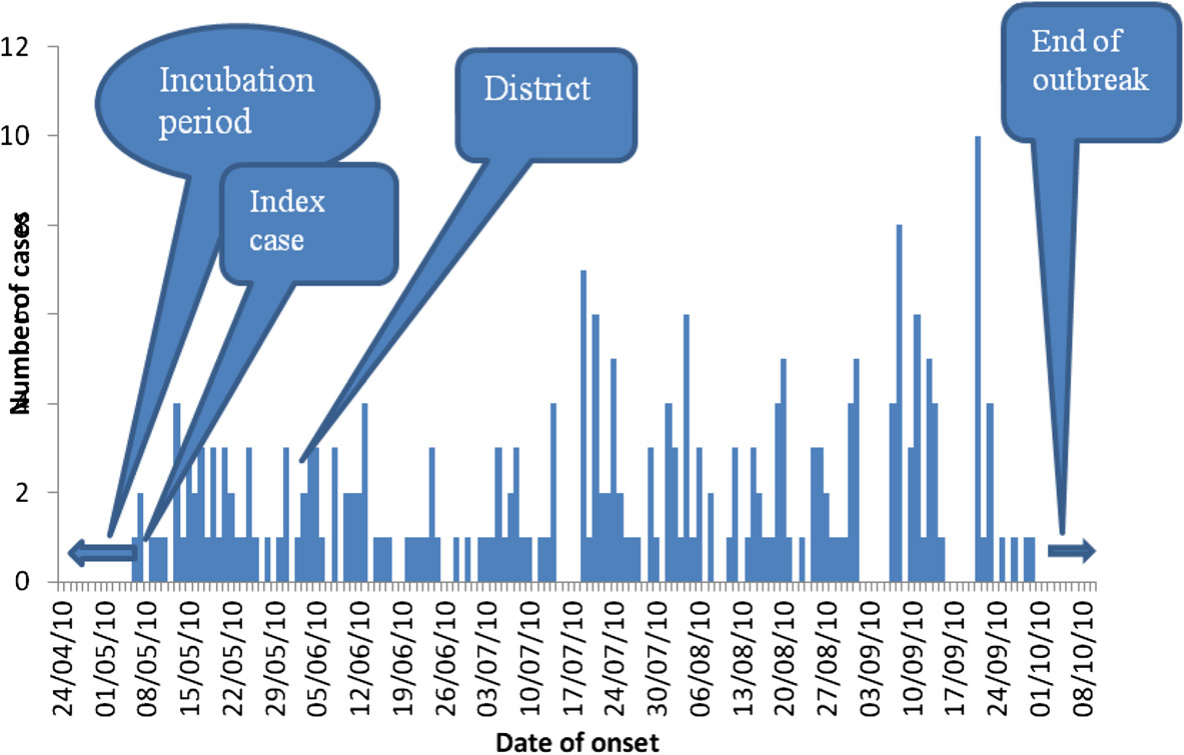
Distribution of measles cases by area of residence, Zaka district 2010.

The median age of cases was 10(Q1=7; Q3=16) years while that of controls was 16 (Q1=10; Q3=19) years. Majority of cases 70 (63.65%) belonged to the apostolic sect and 24(21%) were from orthodox churches as shown in Table 1. The most affected area was Ndanga and the least was Mukanganwi and Chikwanda as shown in Figure 1. The measles vaccination coverage for Zaka district was 75%. Ninety two (83.6%) of cases were not vaccinated and thirty two (29%) of cases were unvaccinated. Zaka district vitamin A supplementation coverage verified by the child health card was at 66.5%.

**Figure 2:**
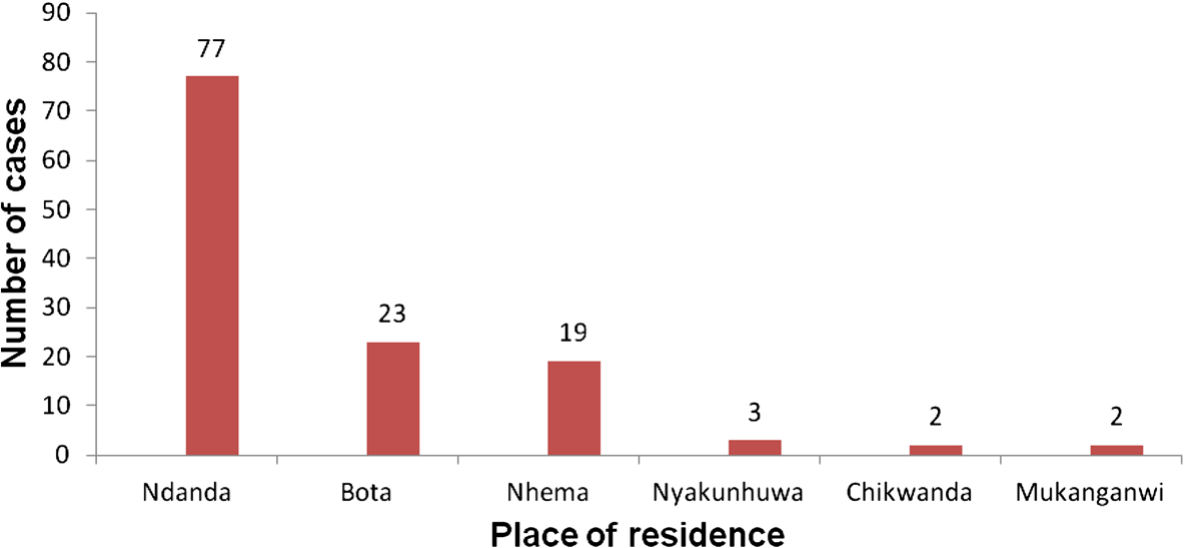
Epi Curve showing the distribution of measles cases by time in Zaka District, 2010.

There were multiple peaks on the Epi curve as shown in Figure 2. The index case had onset of symptoms on the 6th of May. Interventions (treatment of cases, mass vaccinations, health education, advocacy and contact tracing by environmental heath technicians) to the outbreak however started on the 3rd of June 2010 almost a month after the outbreak onset. The outbreak lasted for four months (May to August 2010).

The most reported symptoms were koplik spots 104(94.5%), maculopapular rash 103(93.6%), red eyes 97(88.2%), coryza 55(50.8%), fever 110 (100%) and the least was cough 30 (27.3%). The other symptoms that were reported were vomiting 20 (18.2%), loss of appetite 15(13.6%), and fatigue 40(36.36%).

Out of the 110 cases that were interviewed 45(40.9%) reported seeking medical treatment for measles while 78(70.9%) of controls reported believing in medical treatment for measles. Sixty two (56.4%) of cases were treated for measles at home. Several methods were used for treatment of measles at home and these included herbs 57 (25%), holy water 10 (4.5%), prayer 5 (2.3%) and pain killers 3 (1.4%). There was no significant difference on knowledge of measles between cases and controls.

Okra was the most widely used substance for treatment of measles in Zaka with 57(51.8%) of cases using it. The least used treatment method was pain killers 3(2.7%).

Fifty eight (69.0%) sought treatment at the hospital while 42(31%) were treated for measles at home and 10 cases were treated at school. One hundred (91.8%) of cases sought treatment within three days of onset of illness while the rest of the cases (8.2%) sought treatment later than three days. The median duration for seeking treatment after onset of illness was three days (Q1=2; Q3=7).

In bi-variate analysis, not being vaccinated against measles OR 12.46(95%CI 6.20:25.31) and being below the age of 18 years OR 18.44(95%CI 4.27:79.64) and not believing in medical treatment for measles OR 1.83 (95%CI 1.40:2.40) were significant risk factors for contracting measles. as shown in Table 2.

**Table 2:** Risk factors for contracting measles in, Zaka district, Masvingo Province, 2010

**Exposure**	**Yes/No**	**Cases**	**Controls**	**OR**	**Confidence interval**
History of travel two weeks prior onset of illness	Yes	28	7	5.0	2.10- 12.08
	No	82	103		
Contact with case	Yes	104	12	169	57.73-499.78
	No	6	98		
Being unvaccinated	Yes	92	32	12.46	6.20- 25.31
	No	18	78		
Being under 18 years	Yes	108	82	18.44	4.27- 79.64
	No	2	28		
Not believing in medical treatment for measles	Yes	70	33	4.08	2.32- 7.17
	No	40	77		

In multivariate analysis, factors that remained independently associated with contracting measles in Zaka district measles outbreak were, contact with a case AOR=41.14(95%CI: 7.47-226.54), being unvaccinated AOR= 3.96(95%CI: 2.58-6.08) and not receiving additional doses of vaccine AOR 5.48 (95%CI: 2.16-11.08.) as shown in Table 3.

**Table 3:** Independent factors associated with contracting measles in Zaka district Masvingo Province, 2010

**Exposure/factor**	**AOR**	**95% Confidence interval**	**P value**
Contact with case	41.14	7.47- 226.54	0.000
Being unvaccinated against measles	3.96	2.58-6.08	0.004
Not Receiving additional doses of measles vaccine	5.48	2.16-11.08	0.03

Zaka district had 99% of nurses post filled and every health centre being manned by at least one trained nurse. The District Nursing Officer post, community nurse post, Environmental Health post, pharmacy technician post, health information officer post, health promotion, nutritionist post and the District Medical Officer post were all filled while the laboratory scientist post was vacant. There were 44% environmental health posts filled in Zaka district.

Measles vaccine, thermometers, paracetamol, aspirin, amoxicillin capsules and syrup, tetracycline, oral rehydration solutions, intravenous fluids and needles as well as syringes and vitamin A were available.

Three vehicles were available at the district office and the Expanded Programme on Immunisation truck was in good condition at the time of the outbreak. However fuel was not available at the station. There were no Information Education materials (IEC) on measles readily available.

The district delayed notifying the province of the outbreak. In addition the district took long to do field investigations. The interventions that the district instituted were immunisation of apostolic sect children with the aid of the police and environmental health technicians who had information on sick children in the village and health education.

There was poor surveillance as the district officers did not analyse data and disseminate information. Poor team work was reflected by the failure of the district team to hold outbreak update meetings and some officers lacked current outbreak information like how many cases were recorded.

The most affected area was Danda village followed by Bota and the least was Mukanganwi village as shown Figure 1.

## Discussion

This study identified several factors that were associated with contracting measles in Zaka district. Being unvaccinated against measles was a risk factor for contracting measles. So JS et al. in a measles outbreak investigation in Korea reveal the same finding that cases were high in unvaccinated and incompletely vaccinated children than those who were completely vaccinated [9]. In addition Lack of vitamin A supplementation was also found to be a risk factor contracting measles. Low Vitamin A supplementation in Zaka may lead to increased risk of contracting measles and its complications. This is consistent with findings by Mishra et al., in a measles investigation outbreak in India where the Vitamin A supplementation coverage was less than 30% [10]. Vitamin A supplementation has been shown to increase measles specific antibody formation if it is administered simultaneously with the measles vaccine.

The low immunisation coverages and vitamin A supplementation coverages in Zaka district could be attributed to the huge population of religious objectors to immunisation [3].

There was high case fatality rate in the outbreak. This could be due to the fact that majority of the cases were from the apostolic sect who are against seeking medical treatment. This is in contradiction with findings by Bara et al. who reported a case fatality of 0.6% in a measles outbreak in Epworth [11] However in developing countries case fatalities due to measles are between 3-5% [7]. This high case fatality in Zaka may be attributed to the use of okra to treat measles and at times prayer.

In Zaka, majority of the cases sought treatment for measles at hospital. The apostolic sect members had their own isolation centres were most measles cases were kept. Majority of cases did not believe in seeking medical attention for measles or taking their children for measles vaccination. This was also reported by Qidwai et al [12] who mentioned that factors like knowledge, attitudes and practices of parents affect immunisation campaigns negatively.

Having contact with a measles case was also found to be a risk factor. This is also supported by the ministry of health of Zimbabwe, which states that children who live in crowded places are at high risk of contracting measles, and that a person with measles can infect others for several days before he/ she develops symptoms. Measles spreads easily in places were children gather for example schools [2].

Stein-Zamir et al. in an measles outbreak in ultra-orthodox Jewish community who reported that most cases were from big families and 70% were unvaccinated and below the age of 14 years [13]. A study by Syed M et al. also showed that having more than one child at a house posed as a risk factor to contracting measles [14] This was also reflected in our study that had a median number of siblings of four and the WHO also reports that overcrowding in developing countries is a risk factor for contracting measles [15,16]. Knowledge among cases and controls were not different. This might due to health education that was given during field investigations by health workers.

We therefore concluded that the measles outbreak in Zaka resulted from the existence of a large number of unvaccinated children among religious objectors in the area and low awareness of the disease.

We recommend the promotion of awareness in the community by health education and promotion. District Medical Officer to facilitate formulation of Emergency Preparedness plans (EPR). In the long term we recommend that the Ministry of Health should make it mandatory for all children to be vaccinated before enrolling into primary or boarding schools.

Actions taken so far based on findings of this investigation was training of District Nursing Officers, community sisters and District Environmental Health Officers in EPI disease surveillance and outbreak management in Masvingo province.

## Competing interests

The authors had no competing interests.

## Authors’ contribution

KWP Designed the protocol , collected data , analysed data and wrote the report. RF Assisted in designing protocol, analysis of data, and report writing. NG Assisted in designing protocol, analysis of data, and report writing. All authors read and approved the final manuscript.
